# Characterization of an Additive Manufactured TiAl Alloy—Steel Joint Produced by Electron Beam Welding

**DOI:** 10.3390/ma11010149

**Published:** 2018-01-17

**Authors:** Gloria Basile, Giorgio Baudana, Giulio Marchese, Massimo Lorusso, Mariangela Lombardi, Daniele Ugues, Paolo Fino, Sara Biamino

**Affiliations:** 1 Department of Applied Science and Technology, Politecnico di Torino, Corso Duca degli Abruzzi 24, 10129 Torino, Italy; giorgio.baudana@polito.it (G.B.); giulio.marchese@polito.it (G.M.); mariangela.lombardi@polito.it (M.L.); daniele.ugues@polito.it (D.U.); paolo.fino@polito.it (P.F.); sara.biamino@polito.it (S.B.); 2 Istituto Italiano di Tecnologia (IIT), Center for Space Human Robotics, Corso Trento 21, 10129 Torino, Italy; massimo.lorusso@iit.it

**Keywords:** γ-TiAl alloys, electron beam melting, joining, electron beam welding, nanoindentation

## Abstract

In this work, the characterization of the assembly of a steel shaft into a γ-TiAl part for turbocharger application, obtained using Electron Beam Welding (EBW) technology with a Ni-based filler, was carried out. The Ti-48Al-2Nb-0.7Cr-0.3Si (at %) alloy part was produced by Electron Beam Melting (EBM). This additive manufacturing technology allows the production of a lightweight part with complex shapes. The replacement of Nickel-based superalloys with TiAl alloys in turbocharger automotive applications will lead to an improvement of the engine performance and a substantial reduction in fuel consumption and emission. The welding process allows a promising joint to be obtained, not affecting the TiAl microstructure. Nevertheless, it causes the formation of diffusive layers between the Ni-based filler and both steel and TiAl, with the latter side being characterized by a very complex microstructure, which was fully characterized in this paper by means of Scanning Electron Microscopy, Energy Dispersive X-ray Spectroscopy, and nanoindentation. The diffusive interface has a thickness of about 6 µm, and it is composed of several layers. Specifically, from the TiAl alloy side, we find a layer of Ti_3_Al followed by Al_3_NiTi_2_ and AlNi_2_Ti. Subsequently Ni becomes more predominant, with a first layer characterized by abundant carbide/boride precipitation, and a second layer characterized by Si-enrichment. Then, the chemical composition of the Ni-based filler is gradually reached.

## 1. Introduction

In recent years, the aerospace and automotive industries have been showing great interest in gaining control over polluting emissions in the environment. For this reason, there is the need, as a primary prerequisite, to decrease the weight of components in order to achieve reductions in terms of fuel consumption, emissions and costs. In addition, the decrease in component weight would cause the reduction of centrifugal forces that rotating components are subjected to [1].

In this scenario, the adoption of Titanium Aluminides, i.e., γ-TiAl alloys, as structural materials appear desirable—including for turbine blades, turbocharger wheels and valves—replacing Nickel-based superalloys [2,3,4]. In fact, γ-TiAl alloys show very promising properties, including high specific strength, retention of high specific modulus at elevated temperatures, and good corrosion and oxidation resistance, ensuring a reduction in weight of 50%, with a density of about 4 g/cm^3^ compared to about 8 g/cm^3^ for Nickel-based superalloys [5,6].

Considering these properties, γ-TiAl alloys could compete with superalloys in aerospace and automotive applications. However, the benefits derived from the weight reduction are useless when considering the manufacturing costs associated with the production of TiAl components. In fact, their poor machinability and brittleness make them non-competitive in such applications [4]. This problem has pushed researchers to develop the alternative manufacturing methods Net Shape and Near Net Shape.

Additive manufacturing—in particular Electron Beam Melting (EBM)—seems a promising choice for the production of TiAl [7,8,9,10]. Indeed, this technology allows the production of dense final components, even those of great complexity, reducing most of the post-processing related to traditional manufacturing technologies. A bed of spherical powder with the right thickness is distributed on a platform where an electron beam selectively melts the powder. The component is fabricated layer by layer in this way. In addition, with proper tuning of the process parameters, it is possible to obtain a fully densified part with a homogeneous equiaxed γ-microstructure [10,11]. On the other hand, the EBM manufacturing process is considered a Near Net Shape process due to the external roughness Ra obtained of about 30 µm; thus, the presence of a sufficient overstock material is necessary for subsequent post-EBM machining and finishing processes.

Regarding the automotive field, the main application for Titanium Aluminides is turbocharger wheels, coupled with steel shafts. In the case of turbocharger applications, also, the reduction in weight compared to nickel-based superalloys implies an increase in engine efficiency and, consequently, a reduction in emissions and fuel consumption [12,13].

The joining of such dissimilar materials can be achieved through only a few techniques thus far reported in the literature. Ding J. et al. [14] proposed the direct joining of γ-TiAl turbocharger (Ti-46Al-2Cr-2Nb at %) to a 40Cr steel shaft (Fe-0.4C-1Cr-0.7Mn-0.3Si wt %) with Electron Beam Welding (EBW), trying to obtain a sound joint by modifying the welding parameters, such as heat input. Dong H. et al. [15] studied the joining of γ-TiAl alloy (Ti-43Al-2Cr-Zr-Fe at %) to 42CrMo steel (Fe-0.43C-1.09Cr-0.67Mn-0.31Si-0.2Mo wt %) through direct friction welding, examining the mechanical properties and the obtained microstructure. On the other hand, brazing of TiAl is more difficult than that of common metallic materials, because of its reactivity, which can cause the formation of secondary phases. Noda T. [16] suggested the induction brazing method in an Ar atmosphere as a valid technique for joining the steel shaft to the TiAl turbocharger wheel, with a silver-based alloy as filler material. Tetsui T. et al. [17,18] investigated the joining of a TiAl alloy (Ti-47.1Al-7.8Nb-0.98Cr-0.47Si at %) to a steel shaft, with Incoloy 909 (Fe-38Ni-13Co-2Ti-5Nb wt %) as insert component to mitigate the thermal expansion coefficient between the TiAl alloy and the steel. Specifically, the joint between this insert and the steel rod was realized by EBW, instead of the joint between TiAl and the insert being obtained by brazing. In the latter case, different filler materials were investigated as possible candidates, such as silver, gold, palladium, titanium and nickel alloys.

In this work, a joint trial was realized between a TiAl cylinder (simulating a TiAl turbocharger wheel) and a real CrMo steel shaft. Considering all the above reported findings in the literature about joining, this was accomplished by direct EBW with a nickel-based filler material. The adoption of EBW was dictated by the possibility of heating and melting only the precise location of the filler, thus reducing the deformations of the heat-treated zones and the thermal residual stresses [14,19]. The aim of this work is the characterization of the joint trial from a microstructural point of view, trying to evaluate the diffusive interfaces and new phases generated during the joining procedure.

## 2. Materials and Methods

The TiAl-based alloy used in this work has a nominal chemical composition of Ti-48Al-2Nb-0.7Cr-0.3Si (at %) and is called RNT650 [16]. The TiAl cylinder (10 mm in length and 15 mm in diameter) was produced at the Fraunhofer Institute for Manufacturing and Advanced Material in Dresden by EBM technology using an Arcam A2X machine (Arcam AB, Mölndal, Sweden) [20]. The TiAl part was joined to the steel shaft in the as-EBM condition after proper machining of the part to be welded. The machining was performed in order to produce a cylinder with a low roughness level, fitting the steel junction region, also taking into account the dimensional tolerance necessary to insert the filler material foil.

The shaft material employed was a CrMo steel alloy to JIS SCM435H with nominal composition 0.35C, 1Cr and 0.25Mo (wt %). The steel shaft was received machined to the final configuration for the assembly.

**Figure 1:**
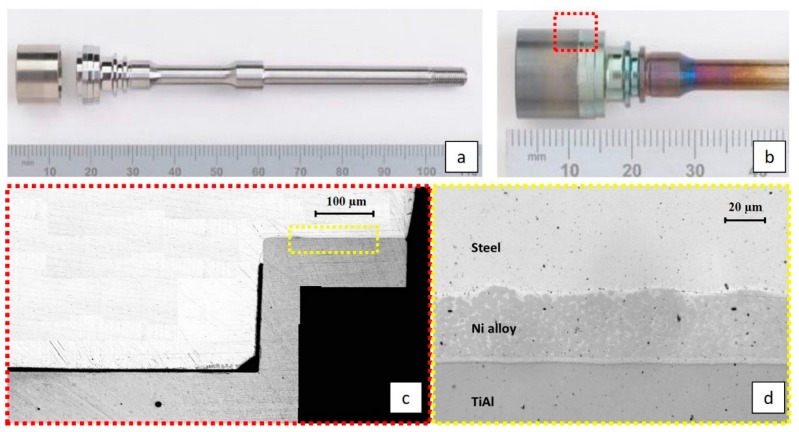
(**a**) TiAl cylinder and Steel shaft before the assembly; (**b**) TiAl cylinder and steel shaft after the assembly; (**c**) longitudinal section of the joint; (**d**) optical microscope image of the joint.

The joint trial was produced, simulating the existing assembly in a real turbocharger, as shown in Figure 1.

The selected filler alloy was the METGLAS^®^ MBF-30; an amorphous nickel-based braze alloy with 4.5Si, 3.2B and 0.06C (wt %). The brazing temperature was >1085 °C, with solidus and liquidus in the range 984–1084 °C, which is suitable for brazing ferrous materials and nickel alloys [21]. The nickel alloy, in the form of 40 µm foil sheet, was delivered to TWI Ltd (Great Abington, Cambridge, UK), who laser cut the foil to adapt it to the cylinder and the shaft, and executed the welding of the three materials using a Hamilton Standard high-voltage EB welding machine (PTR, Enfield, CT, USA). The welding was performed using a localized, diffuse heat source provided by a defocused electron beam positioned either side of the filler line, thereby locally heating the steel and TiAl parts above the filler liquidus, melting the Ni foil by conduction and forming a joint at the planar interface at the end of the rotor wheel. The EBW process was carried out by TWI Ltd.

The evaluation of the joint consisted of microstructural observation with an optical microscope (Leica DMI5000 M, Leica, Wetzlar, Germany), Scanning Electron Microscope (SEM) (Phenom XL, Phenom-World BV, Eindhoven, The Netherlands) with a fully integrated Energy Dispersive X-ray Spectroscopy (EDS) detector, and Field Emission Scanning Electron Microscope (FESEM) (Zeiss Merlin, Zeiss, Oberkochen, Germany) equipped with an INCA Oxford EDS detector. The samples for microstructural analysis were previously ground with SiC papers and then polished with diamond paste up to 1 µm. For the TiAl microstructural evaluation, Kroll′s etching was used. The nanomechanical properties of the joint were determined through nanoindentation measures (Hysitron TI 950 Triboindenter, Hysitron-Bruker, Minneapolis, MN, USA). The diamond indenter was a Cube Corner tip with a low load of 0.4 mN and a dwell time of 5 s; in this way, it was possible to reduce the distance between each indentation up to 0.5 µm, and realize a 17 × 33 grid. 

## 3. Results and Discussion

The primary observation of the joint with the optical microscope allowed the verification of the nature of the adhesion between the three materials, revealing an interdiffusion zone that was very complex with regard to phase formation. A promising quality of the joint was observed, in that there were no cracks, and only a limited number of voids and pores that could be reduced or even eliminated through process optimization.

**Figure 2:**
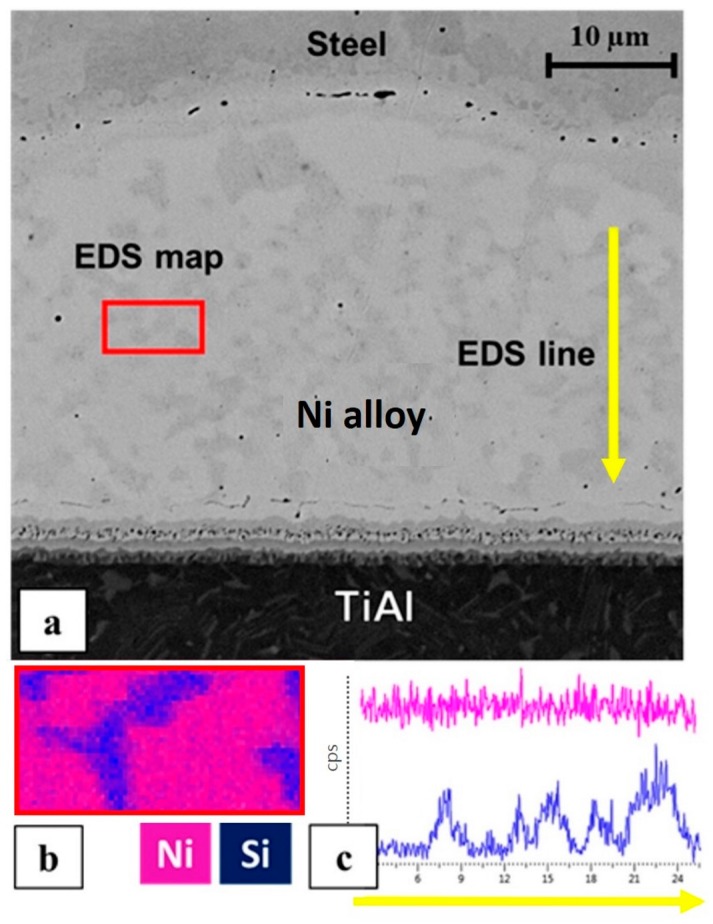
(**a**) back-scattered electrons image of the joint; (**b**) EDS map; (**c**) EDS line.

The optical microscope observation (Figure 1d) evidenced an inhomogeneity in the filler layer, showing brighter areas within the filler material and at the interfaces with the TiAl and steel. The observation of the sample with SEM (Figure 2a) confirmed this inhomogeneity; specifically, the EDS line and EDS map analyses (Figure 2b,c) highlighted the presence of silicon-rich areas, which are responsible for the darker spots in the intermediate Ni layer.

**Figure 3:**
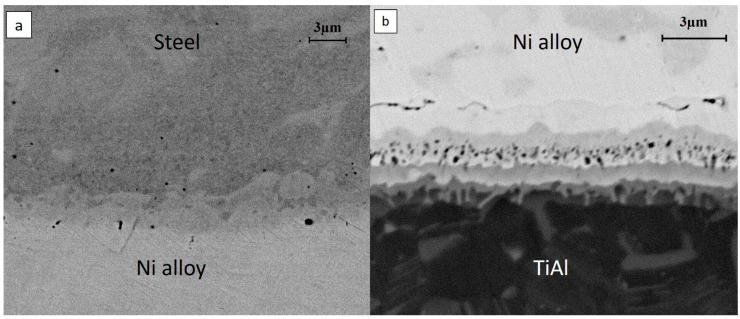
Diffusive interfaces between (**a**) steel and nickel-based material; and (**b**) TiAl and nickel-based material.

The higher magnification back-scattered electron image of the junction (Figure 3) indicated the diffusive interfaces generated during the joining procedure. On the side of the steel (Figure 3a), it was not possible to observe the formation of any new phases during the welding procedure, there was only a gradient of elements at the interface between the Ni alloy and the steel. In contrast, a very complex interface was detected at the diffusive interface between TiAl and the Ni-based alloy (Figure 3b), as previously demonstrated in the works of Tetsui T. [17] and Liu J. et al. [22], where they bonded a TiAl alloy to a nickel-based filler material and pure nickel, respectively. As a result of solid-state diffusion, several layers were produced, with thicknesses of about 6 µm.

To better understand the nature of the observed phases and the distribution of the single elements, punctual, linear and map EDS and nanoindentations were executed across the diffusive interface between the TiAl and the Ni alloy. 

**Figure 4:**
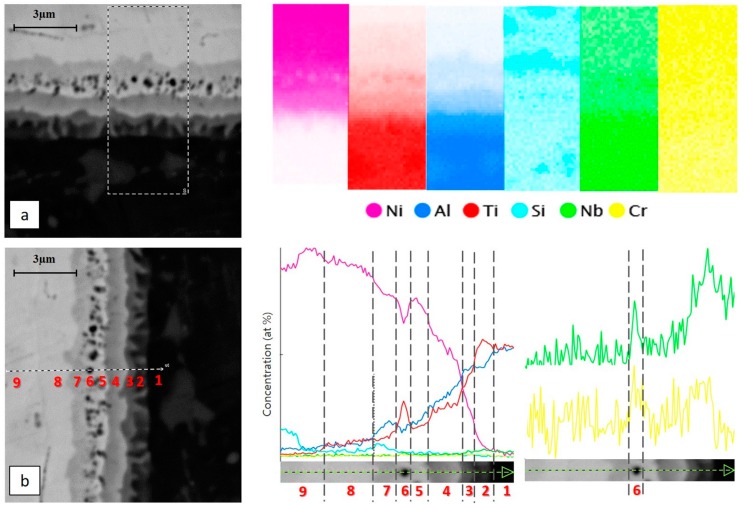
(**a**) EDS map along the TiAl-Ni base filler interface; (**b**) EDS line.

Starting from the unaltered TiAl RNT650 alloy (labelled with the number 1 in Figure 4) it is possible to recognize a continuous grey layer, mainly inside the brighter saw-tooth profile. The grey layer and the brighter saw-tooth one are indicated with the numbers 2 and 3, respectively.

**Figure 5:**
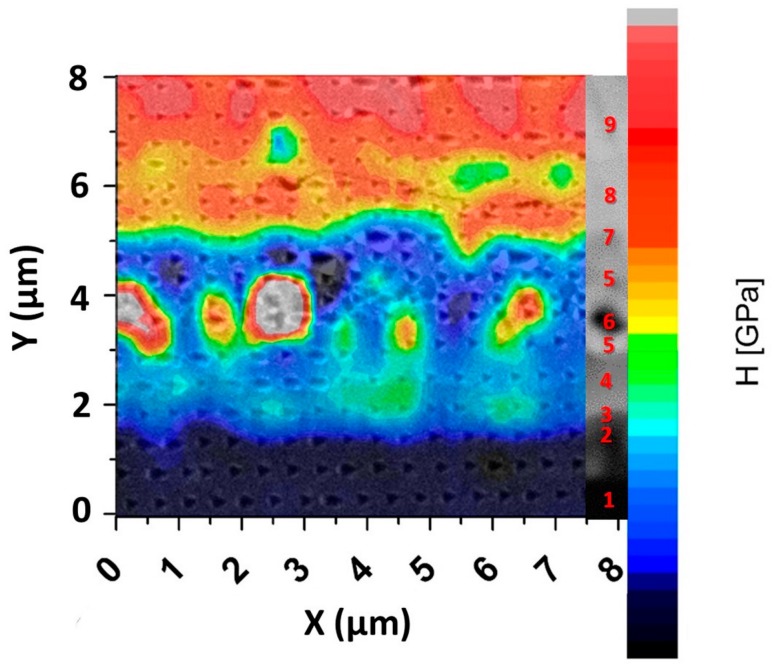
Nanoindentation analysis at the TiAl-Ni interface.

Layer number 2 can be attributed to the Ti_3_Al alloy since, as shown in Figure 4b, an enrichment in titanium was recorded with respect to the base alloy. In contrast, the nickel, coming from the Ni-based filler, could only be detected in very limited amounts. The presence of this phase was confirmed by the nanoindentation map (Figure 5); in fact, this intermetallic corresponds to an increase of hardness [23]. In layer number 3, which is the brighter one, with fringes penetrating into layer number 2, the EDS analysis pointed out a still-high level of nickel concentration, coupled to the presence of titanium and aluminum. The fringes could be due to a non-homogeneous diffusion of nickel through the RNT650 biphasic alloy during the welding procedure. In the work of Liu J. et al. [22], when joining TiAl alloy to Ti_3_SiC2 with pure nickel brazing material, they found a similar morphology, which they identified as Al_3_NiTi_2_. In our case, too, through the EDS line (Figure 4b) and the punctual EDS, an approximate atomic proportion of 3:1:2 was estimated between Al:Ni:Ti, which corresponds to Al_3_NiTi_2_.

Subsequently, a continuous thicker grey layer (labelled with the number 4 in Figure 4) was detected. Here, nickel was more abundant than titanium and aluminum. For this layer, EDS pointed out the following average composition: 26.7 at % for aluminum, 22.6 at % for titanium and 48.4 at % for nickel; therefore, this phase was able to be associated to AlNi_2_Ti.

The chemical compositions given for layers 3 and 4—Al_3_NiTi_2_ and AlNi_2_Ti, respectively—were only average compositions, because these layers corresponded to a range of compositions, due to the diffusion of the elements during the EBW process, as can be seen in the line profiles given in Figure 4b, which shows the thickness of these layers. However, this is in good agreement with the Ti-Al-Ni ternary alloy phase diagram, in which they have a large composition range [24].

Continuing to approach to the Ni-based filler, a continuous white layer with black particles disseminated throughout was detected. The white layer is labelled with the number 5 in Figure 4, while the black particles are identified with number 6. In layer 5, an abundance of nickel (Figure 4a,b) was detected, thus suggesting that the filler material had been reached, here. Nevertheless, the diffusion of the elements from the TiAl alloys, such as titanium, chromium and niobium, that reached the carbon and the boron contained in the filler induced the precipitation of carbides and borides (the black particles). The EDS map in Figure 4a and the EDS lines in Figure 4b confirm that those particles were enriched in titanium, chromium and niobium. This finding is also consistent with the literature, in which Tetsui T. [17] identified these precipitates as borides, and Liu J. et al. [22] as carbides. The ceramic nature of these boride and carbide phases was able to be confirmed by nanoindentation. The analysis showed hard precipitates in the diffusive layer (Figure 5, grey and red dots) compatible with borides or carbides [25,26].

For the grey continuous layer (labelled with number 7 in Figure 4) a high concentration of silicon (Figure 4a,b) was measured. Silicon may come from both TiAl alloy and nickel alloy but, considering the concentration, it most likely came from the nickel alloy. According to Liu J. et al. [22], silicon displays a high migration activity; for this reason, it was able to actively participate in promoting the bonding of materials reacting with the other elements. With regard to the nanoindentations (Figure 5), the high silicon concentration in this layer could cause an increase in hardness, in comparison to the previous layers. 

Finally, it was possible to observe a lighter grey continuous layer (number 8 in Figure 4), where there were some traces of Ti and Al diffusion, but the composition started approaching the composition of the nickel alloy. The nickel-based material was finally reached in the next zone (number 9 in Figure 4), whose composition corresponded to that reported previously (see Figure 2). In Figure 4a,b or Figure 5, it is possible to appreciate the silicon-rich regions discussed previously. In particular, the nanoindentation showed higher values of hardness for these zones, in accordance with the increased hardness in the layer labelled with number 7, which was also rich in silicon.

**Figure 6:**
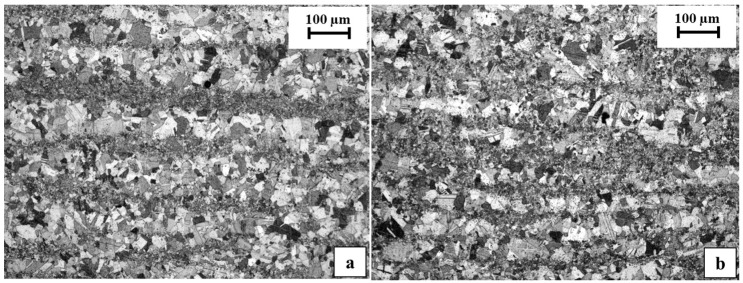
TiAl microstructure (**a**) near the diffusive interface with the nickel-based alloy; (**b**) far from the interface.

In order to evaluate the microstructure of the TiAl part and to check whether it was ultimately affected by the temperature reached during welding, the polished specimen was etched with Kroll’s solution and analyzed by optical microscopy. The resulting microstructure was an equiaxed gamma microstructure, as was expected for the as-EBM TiAl material [10,11,27]. However, a layered appearance of the microstructure, in which bigger and smaller grains were alternated, was observable (Figure 6), which would be worth avoiding through fine tuning of the process parameters of the Electron Beam additive manufacturing process [11,27]. Nonetheless, for the purposes of the present work focused on joining, a fact that is interesting to point out emerges when comparing the microstructure in an area close to the junction region (Figure 6a) with the microstructure observed in another area of the specimen far from the junction (Figure 6b). No microstructural modification due to the temperatures reached during the joining process could observed, and therefore, microstructure was not affected during the welding.

However, the observed as-EBM gamma microstructure is not a desirable one with regard to its corresponding mechanical features. In order to obtain a particular microstructure leading to improved mechanical properties for the turbocharger application, a post-processing heat treatment will be necessary for the TiAl part. As reported in the work by Baudana G. et al. [10], a near lamellar microstructure makes it possible to reach the desired mechanical behaviors, such as creep resistance and tensile properties. It is important to underline that it would be necessary to heat-treat the TiAl part prior to the joining process in order to avoid submitting the entire junction to heat treatments at high temperatures.

## 4. Conclusions

This study was an initial investigation of the feasibility of a joint trial between TiAl and steel parts using a Ni alloy as filler material. The following conclusions can be deducted:

- A promising quality of the joint was observed by optical and electronic microscopy, in that there were no cracks, and only a limited number of voids, which an optimization of the joining process parameters could reduce or even eliminate.
- The microstructure observed in the TiAl part of the joint was the typical EBM equiaxed gamma microstructure, meaning that the EBW process did not affect the TiAl microstructure. It is necessary to point out that these joints were produced using the TiAl part in its Electron Beam additive technology condition. For the investigated applications, it would be necessary to produce the joints using a heat-treated TiAl part and heat-treated steel shaft, because performing the heat treatment after the joining process would affect the properties of the different parts of the assembly.
- The welding process causes the formation of diffusive layers between both Ni and steel and Ni and TiAl, with the latter side being characterized by a very complex microstructure, which has been fully characterized in this paper.

Further studies must be focused on the mechanical characterization of these joints.
